# Association between Oral Health Status and Diabetic Nephropathy-Related Indices in Japanese Middle-Aged Men

**DOI:** 10.1155/2020/4042129

**Published:** 2020-06-07

**Authors:** Masami Yoshioka, Yoshifumi Okamoto, Masahiro Murata, Makoto Fukui, Shizuko Yanagisawa, Yasuhiko Shirayama, Kojiro Nagai, Daisuke Hinode

**Affiliations:** ^1^Department of Oral Health Sciences, Faculty of Health and Welfare, Tokushima Bunri University, 180 Nishihamaboji Yamashirocho Tokushima, 770-8514, Japan; ^2^Tokushima Dental Association, 1-8-65 Kitatamiya, Tokushima 770-0003, Japan; ^3^Tokushima University Graduate School of Biomedical Sciences, 3-18-15 Kuramotocho, Tokushima 770-8504, Japan

## Abstract

Oral health status is known to be associated with lifestyle-related diseases such as diabetes and chronic kidney disease. In Japan, around 40% of hemodialysis cases are patients with diabetic nephropathy. The aim of this study was to clarify the association between oral health status and diabetic nephropathy-related indices in Japanese middle-aged men. Sixty-six men (age range: 55–64 years) with ≥20 remaining teeth and who received public medical checkups and oral examinations were enrolled. We examined correlations of age, body mass index, HbA1c, HDL-C, LDL-C, neutral fat, serum creatinine, and the estimated glomerular filtration rate (eGFR) with the number of remaining teeth or the community periodontal index (CPI) score (periodontal pocket < 4 mm: 0, 4–6 mm: 1, ≥6 mm: 2). A positive correlation between the CPI score and serum creatinine and a negative correlation between CPI score and eGFR (Spearman's rank correlation coefficient, *r* = 0.459, *p* < 0.01, and *r* = −0.460, *p* < 0.01, respectively) were observed. The mean eGFR in the CPI score 0 group was significantly higher than that in the CPI score 1/2 group (82.6 vs. 70.7, Student's *t*-test, *p* < 0.01). Logistic regression analysis using eGFR as a dependent variable and age, CPI score, body mass index, HbA1c, and neutral fat as independent variables suggested that low eGFR (<60) could be attributed to CPI score (OR = 3.169, 95% CI: 1.031–9.742, *p* = 0.044). These results suggest a possible association between periodontal status and renal function in Japanese middle-aged men. Periodontal condition is controlled by oral prophylaxis, and periodontal disease and chronic kidney disease have some common risk factors. Thus, periodontal management can contribute to the prevention of severe chronic kidney disease.

## 1. Introduction

The number of Japanese patients with progressive chronic kidney disease (CKD) on hemodialysis has increased [1]. Because around 40% of cases are due to diabetic nephropathy, prevention of severe diabetic nephropathy is a crucial issue [1]. In Japan, a nationwide screening program was initiated in 2008 (the Specific Health Checkups and Specific Health Guidance program), which targets people aged 40–74 years to detect those with metabolic syndrome and to offer them lifestyle services [2]. This program is expected to reduce the prevalence of lifestyle-related diseases including diabetes [3].

The prevalence of CKD in the middle-age group is higher in men than in women among the Japanese general population [4]. According to the Annual Dialysis Data Report 2016 by Japanese Society for Dialysis Therapy, the most common 5-year age group among incident patients was 65-69 years for men, and the prevalent dialysis patient was higher in middle-aged men compared with middle-aged women [5].

Previous studies have suggested that periodontal disease is a risk factor that worsens glycemic control and causes deterioration of renal function [6, 7]. There are a few epidemiological studies in Japan which have detected a relationship between periodontal status and renal function. And they focused on older subjects or women, not on middle-aged men [8, 9].

Taken together, to clarify an association between periodontal disease and diabetic nephropathy-related indices in Japanese middle-aged men would provide useful information for enlightenment activities towards the middle-aged men. We conducted the present study to test the hypothesis that the severity of periodontal condition and/or the number of remaining teeth might be associated with diabetic nephropathy-related indices in Japanese middle-aged men.

## 2. Materials and Methods

Sixty-six men (age range: 55–64 years) with ≥20 remaining teeth, who received public medical checkups and oral examinations during 2013–2018 at Anan City, Tokushima Prefecture, Japan, were included in this observational study. Oral examination including periodontal disease screening was performed at the municipality level by 8 well-trained dentists according to the manual for periodontal disease screening published by the Ministry of Health, Labor and Welfare of Japan [10]. Oral examination was conducted under sufficient lighting in a private room at the public medical checkups site, and periodontal examination was performed using the World Health Organization (WHO) probe, as also known as the CPI probe. We obtained a medical examination data set (age, body mass index (BMI), HbA1c, HDL-C, LDL-C, neutral fat, serum creatinine, and the estimated glomerular filtration rate (eGFR)) combined with oral examination data (the number of remaining teeth and the community periodontal index score (CPI score; periodontal pocket < 4 mm: 0, 4–6 mm: 1, ≥6 mm: 2)). And then, we investigated correlations of medical examination parameters and oral examination parameters. These medical parameters are all measurement item of Specific Health Checkup towards 40-74 years in Japan. Since we needed to analyze medical records related to diabetic nephropathy, we excluded persons who had even one missing measurement.

To find an association between diabetic nephropathy-related factors and CPI score or the number of remaining teeth, Spearman's rank correlation coefficient test was performed. After the Shapiro–Wilk test to check a normal distribution, Student's *t*-test or the Mann–Whitney *U* test was used to compare the medical records between the two groups, according to the CPI score (0 group and 1/2 group). To elucidate factors associated with lower eGFR, binominal logistic regression analysis was used. Statistical analyses were performed using IBM SPSS 22.0 (IBM Inc., Tokyo, Japan), and statistical significance was set at a level of <0.05. This study was approved by the ethics committee of Tokushima Bunri University (No. R1-27).

## 3. Results

### 3.1. Characteristics of Study Subjects

The characteristics of age and medical history in study subjects are shown in Table 1. In the Shapiro–Wilk test, a normal distribution was confirmed for LDL-cholesterol and eGFR among the indicated variables. Therefore, Student's *t*-test was used for these variables, and the Mann–Whitney *U* test was used for the remaining variables.

The distributions of study subjects by the CPI score or eGFR are shown in Tables 2 and 3. As shown in Table 2, almost 70% of the study subjects recorded a CPI score of 1 or 2, which indicates periodontal pockets ≥ 4 mm.

Among 66 subjects, 10 subjects (15.2%) recorded an eGFR lower than 60 ml/min/1.73 m^2^, which is a reference value for stage G3a in CKD.

### 3.2. Association between Diabetic Nephropathy-Related Indices and CPI Score or Number of Remaining Teeth

**Figure 1:**
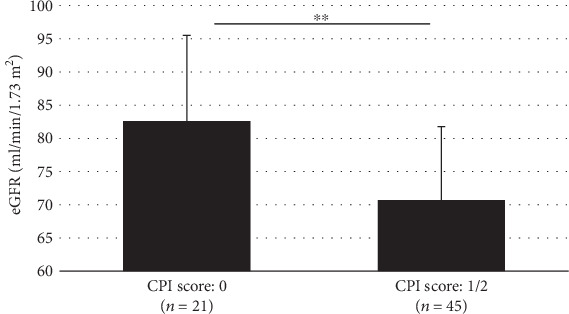
Comparison of eGFR between the CPI score 0 group and the CPI score 1/2 group (Student's *t*-test, ^∗∗^*p* < 0.01).

Spearman's rank correlation coefficient test revealed a positive correlation between the CPI score and serum creatinine and a negative correlation between the CPI score and eGFR (Spearman's rank correlation coefficient, *r* = 0.459, *p* < 0.01, and *r* = −0.460, *p* < 0.01, respectively) (Table 4). The mean eGFR in the CPI score 0 group was significantly higher than that in the CPI score 1/2 group (82.6 vs. 70.7, Student's *t*-test, *p* < 0.01) (Figure 1).

### 3.3. Factors Associated with Low eGFR

We performed binominal logistic regression analysis using eGFR < 60 as the outcome variable and age, CPI score, BMI, HbA1c, and neutral fat as independent variables. The results showed that the CPI score was associated with eGFR < 60 (Table 5).

## 4. Discussion

In Japan, measures to identify metabolic syndrome have been promoted by the Specific Health Checkups and Specific Health Guidance program for the last decade. People aged 40–74 years are required to undergo a medical checkup once a year. For those suspected of having diabetes, a medical consultation is recommended, and health guidance is provided by a public health nurse. Periodontal disease screening is performed at the municipality level; however, it is not mandatory, and the rate of consultation was estimated to be only 4.3% in 2015 [11]. The consultation rate of middle-aged men is relatively low; therefore, we conducted statistical analysis by summarizing the data of subjects for 6 years.

In this study, we targeted people who had periodontal disease screening, so the subjects were limited to those with teeth. The analysis target was limited to those with ≥20 remaining teeth, in order to analyze it together with the periodontal disease index (the CPI score). In general, as periodontal disease progresses and teeth are lost, the CPI score may decrease as a result of the loss of the teeth targeted for periodontal disease screening. Because this study targeted people aged 55–64 years, most had more than 20 teeth, and only one person was excluded because of having fewer than 20 teeth.

A previous study conducted with diabetes outpatients suggested possible relationships between the number of teeth and HbA1c or serum HDL-C [12]. We also found a significant association between the number of remaining teeth and HbA1c or serum HDL-C in this study, which was limited to subjects with ≥20 remaining teeth. In Japan, the 8020 Movement has been developed over the last three decades, which advances the concept that maintaining more than 20 teeth at 80 years of age leads to a healthy and long life. From the perspective of preventing metabolic syndrome, it might be necessary to set goals higher than 20 teeth.

In this study, we analyzed which factors would contribute to eGFR <60 mL/min/1.73m^2^ to find preventive measure against impairment of renal function. According to the CKD Risk Classification and the Classification of Diabetic Nephropathy CKD in Japan, eGFR < 60 mL/min/1.73m^2^ is the cut-off value of Stage G3a [13]. At the Specific Health Checkups towards 40-74 years in Japan, CKD was defined as either the presence of proteinuria or eGFR < 60 mL/min/1.73m^2^ [14]. More than 80% of the individuals defined as CKD corresponded to CKD stage G3 and more, i.e., eGFR < 60 mL/min/1.73m^2^ [4]. Many epidemiological studies related to CKD use eGFR < 60 mL/min/1.73m^2^ as a standard level for lower eGFR [14, 15] A longitudinal 10-year follow-up study in the Japanese general population revealed that lower GFR was found to be a significant risk factor for a faster decline of GFR [16]. Taken together, we defined a low eGFR with a cut-off value of 60 mL/min/1.73m^2^.

Akar et al. reviewed the possible contribution of poor oral health to systemic consequences including infectious diseases, atherosclerotic complications, and protein-energy wasting in patients with CKD [17]. Kshirsagar et al. suggested that initial and severe periodontal disease was associated with a GFR of less than 60 mL/min/1.73m^2^ compared with healthy/gingivitis (OR, 2.00; 95% confidence interval, 1.23–3.24) [18]. Furthermore, several clinical studies support the view that periodontal treatment has a statistically significant positive effect on eGFR [19, 20]. This study suggested that low eGFR might be attributed to periodontal disease in Japanese middle-aged men. Compared with middle-aged women or older men, middle-aged men recorded a lower consultation rate for dental checkups, which indicates a low priority for oral health issues [21]. At a medical checkup that is mandated for everyone, a recommendation for receiving periodontal screening and health education concerning an association between oral health and general health might be effective in preventing diabetes and its complications.

Our study has several limitations. First, persons with less than 20 present teeth were excluded, and findings from this study might not be generalizable to all persons of the same age group. Second, findings from this study might not be generalizable outside of Japan because the prevalence of CKD might be diverse by country [4]. Third, other risk factors such as hypertension and proteinuria are not taken into account in this study.

By the way, we could not calculate the sample size before starting this study, because of lacking previous research conducted with the same age group of men. In this study, we compared the mean eGFR between the CPI score 0 group and the CPI score 1/2 group using Student's *t*-test. Effect size (Hedges' *g*) was -1.02, when calculating from the sample size, mean, and standard deviation (SD) of eGFR in these two groups (21, 82.6, and 12.9 in the CPI score 0 group and 45, 70.7, and 11.1 in the CPI score 1/2 group, respectively) The required sample size calculated using a significance level of 0.05, a power level of 0.80, and an effect size (Hedges' *g*) of -1.02 was 16 in each group for a total of 32. Therefore, we consider the sample size in this study was enough.

A previous epidemiological study revealed that the prevalence of CKD stage G3 and more (GFR < 60 mL/min/1.73 m^2^) increased with age in Japan [4]. In order to prevent the severity of the CKD, it might be important to reduce risk by intervention with changeable factors towards a younger age group before renal function declines when aging. In case this study suggested that the possibility on oral health management might be advantage on suppression of reduced renal function, it might be the novelty of the present work.

In Japan, the government has focused on the prevention of diabetic nephropathy to slow the increase in the number of patients undergoing dialysis [3]. In that initiative, the guidelines for the management of periodontal disease by dental professionals are clearly written. The findings from this study can be used for health promotion and can be provided to medical staff to help improve the understanding of comprehensive dental care.

## 5. Conclusions

Periodontal status may be related to renal function in Japanese middle-aged men. The number of remaining teeth may be related to HbA1c or dyslipidemia. Periodontal condition can be controlled by oral health management. Thus, periodontal examinations and oral health guidance should be included in annual public medical checkups to prevent diabetes-related nephropathy.

## Figures and Tables

**Table 1:** Characteristics of study subjects (*n* = 66).

	Min	Max	Mean	SD
Age	55	64	61.6	2.5
Number of remaining teeth	20	32	26.8	2.5
Body mass index (kg/m^2^)	18.4	38.0	24.1	3.5
Serum creatinine (mg/dl)	0.59	1.20	0.83	0.14
HbA1c (%)	4.9	9.8	5.84	0.79
Estimated GFR (ml/min/1.73 m^2^)	47	108	74.5	12.9
Serum HDL cholesterol (mg/dl)	31	107	59	16
Serum LDL cholesterol (mg/dl)	61	202	127	27
Neutral fat (mg/dl)	35	497	135	77

**Table 2:** Distribution of study subjects by CPI score (*n* = 66).

	*n*	%
CPI: 0	21	31.8
CPI: 1	27	40.9
CPI: 2	18	27.3

**Table 3:** Distribution of study subjects by eGFR (*n* = 66).

	*n*	%
90≦	7	10.6
60-89	49	74.2
≦59	10	15.2

**Table 4:** Spearman's correlation coefficients (*r*) between age and factors associated with metabolic syndrome and CPI score or number of teeth (*n* = 66).

	CPI score (0, 1, 2)	Number of remaining teeth
Age	-0.005	-0.052
	0.969	0.681
Body mass index	-0.031	-0.160
	0.805	0.198
Serum creatinine	**0.459**	-0.008
	**0.000**	0.949
HbA1c (%)	-0.001	**-0.300**
	0.993	**0.014**
Estimated GFR	**-0.460**	0.009
	**0.000**	0.945
Serum HDL cholesterol	0.090	**0.289**
	0.474	**0.019**
Serum LDL cholesterol	-0.170	0.032
	0.173	0.796
Neutral fat	-0.219	-0.034
	0.078	0.784

Upper: Spearman's correlation coefficients (*r*). Lower: *p* value.

**Table 5:** Factors associated with low eGFR (<60 ml/min/1.73m^2^) according to binomial logistic regression analysis (*n* = 66).

Variable	OR	95% CI	*p* value	
Age	1.493	0.902-2.469	0.119	
CPI (0, 1, 2)	**3.169**	**1.031-9.742**	**0.044**	
Body mass index	0.885	0.651-1.203	0.434	
HbA1c	0.423	0.071-2.527	0.346	
Neutral fat	1.005	0.994-1.016	0.350	

Binomial logistic regression analysis was conducted using each of five variables as the dependent variable.

## Data Availability

The data from medical and dental records used to support the findings of this study have not been made available because of ethical concerns to protect the privacy of community-dwelling individuals.
